# Preoperative Glycemic Status in Elective Cardiac Surgery Procedures: HbA1c Independently Predicts Low Cardiac Output Syndrome and Mortality

**DOI:** 10.3390/diagnostics16040515

**Published:** 2026-02-09

**Authors:** Fotini Ampatzidou, Serafeim-Chrysovalantis Kotoulas, Maria Papaioannou, Christina Mouratidou, Eleni Massa, Eleni Mouloudi, George Drossos

**Affiliations:** 1 Cardiac Surgery Intensive Care Unit Department, Georgios Papanikolaou Hospital, 57010 Thessaloniki, Greece; fampatzidou@gmail.com; 2 Department of Adult Intensive Care Unit, Hippokration General Hospital, 49, Konstantinoupoleos Street, 54642 Thessaloniki, Greece; 3 1st Intensive Care Unit, Georgios Papanikolaou Hospital, 57010 Thessaloniki, Greece; mariappnn@yahoo.com; 4 Cardiac Surgery Department, Georgios Papanikolaou Hospital, 57010 Thessaloniki, Greece

**Keywords:** HbA1c, elective cardiac surgery, thoracic aortic surgery, prognostic value, perioperative period, low cardiac output syndrome, mortality

## Abstract

**Background**: The prognostic value of preoperative hemoglobin A1c (HbA1c) in patients undergoing cardiac surgery remains uncertain. This study investigated the association between preoperative HbA1c levels and the risk of postoperative low cardiac output syndrome (LCOS) and in-hospital mortality in patients undergoing elective cardiac and/or thoracic aortic surgery. **Methods**: This single-center retrospective cohort study included consecutive adult patients who underwent elective cardiac and/or thoracic aortic surgery between 1 November 2019 and 30 June 2021. Patients younger than 18 years, pregnant, or lacking a preoperative HbA1c measurement within one week before surgery were excluded. A total of 728 patients were analyzed. Baseline clinical characteristics, operative variables, and postoperative outcomes were collected. Associations between HbA1c and perioperative parameters were assessed using univariate analyses, and independent predictors of LCOS and in-hospital mortality were identified using multivariate logistic regression. **Results**: Higher HbA1c levels were associated with greater body mass index (BMI; r = 0.08, *p* = 0.025), higher New York Heart Association (NYHA) functional class (III vs. I–II: 6.86 ± 1.60% vs. 6.15 ± 1.13%, *p* = 0.001), postoperative LCOS (7.05 ± 1.72% vs. 6.15 ± 1.13%, *p* = 0.013), and in-hospital mortality (6.79 ± 1.50% vs. 6.15 ± 1.13%, *p* = 0.011). In multivariate analysis, HbA1c independently predicted LCOS (odds ratio [OR] 1.52; 95% confidence interval [CI] 1.09–2.12; *p* = 0.015), together with reduced left ventricular ejection fraction and postoperative atrial fibrillation. Independent predictors of in-hospital mortality included BMI, HbA1c (OR 1.81; 95% CI 1.19–2.74; *p* = 0.005), EuroScore II, prolonged cardiopulmonary bypass time, impaired glomerular filtration rate, postoperative septicemia, continuous renal replacement therapy, re-intubation, and prolonged mechanical ventilation. **Conclusions**: Higher preoperative HbA1c levels were independently associated with an increased risk of postoperative LCOS and in-hospital mortality in patients undergoing elective cardiac and/or thoracic aortic surgery. These findings support the role of HbA1c as a prognostic biomarker and its potential integration into preoperative risk stratification models for cardiac surgery.

## 1. Introduction

Cardiac surgery is a highly specialized field that focuses on the treatment of structural and functional heart and thoracic aortic disorders. It includes a wide range of procedures, such as coronary artery bypass grafting, surgical revascularization for ischemic heart disease, valve repair or replacement, heart transplantation, and the surgical treatment of congenital heart disease and cardiac arrhythmias [1]. Cardiopulmonary bypass (CPB) is used in the majority of cardiothoracic procedures to provide a controlled operating environment by temporarily ceasing heart activity and externally supporting circulatory and respiratory systems.

However, CPB is associated with several adverse physiological effects, including systemic inflammatory activation, endothelial dysfunction, and organ hypoperfusion [2,3]. Patient morbidity and mortality are still greatly impacted by postoperative complications such as low cardiac output syndrome (LCOS), acute renal injury, and infectious events, despite significant advances in surgical procedures, anesthesia, and perioperative care [4]. Thus, precise preoperative risk stratification is crucial for enhancing patient selection, directing perioperative management, and elevating postoperative outcomes.

Hemoglobin A1c (HbA1c) widely reflects average blood glucose levels over the preceding two to three months and is used for diagnosing and monitoring diabetes mellitus [5]. Elevated HbA1c has become a surrogate marker of chronic metabolic dysregulation, systemic inflammation, endothelial dysfunction, and impaired microvascular function in addition to its role in glycemic assessment. These pathophysiological processes may adversely impact wound healing, myocardial performance, and susceptibility to postoperative complications [6].

Elevated preoperative HbA1c levels have been associated in several studies with unfavorable outcomes following heart surgery, such as myocardial damage, prolonged mechanical ventilation, sternal wound infections, and higher mortality. However, the available evidence remains heterogeneous. While some investigations demonstrate a significant relationship between HbA1c and postoperative morbidity or mortality, others fail to confirm these associations after adjustment for established surgical risk scores, perioperative glycemic control, and relevant comorbidities [7]. Clarifying the predictive value of HbA1c is especially important in elective cardiac and thoracic aortic surgery, as it represents a potentially modifiable preoperative risk factor.

The independent association between preoperative HbA1c and postoperative LCOS, a significant complication following cardiac surgery and a key factor in adverse outcomes, remains inadequately defined despite its potential clinical importance.

Moreover, the incremental prognostic value of HbA1c beyond established risk stratification tools, such as EuroScore II, remains uncertain. The association between chronic glycemic status and significant postoperative complications, such as acute kidney injury, sepsis, and respiratory failure, requires additional research in large real-world cohorts [8,9,10,11,12].

The objective of this retrospective cohort study was to assess the relationship between preoperative HbA1c levels and the risk of postoperative LCOS and in-hospital mortality in patients undergoing elective cardiac and/or thoracic aortic surgery.

We also sought to ascertain whether HbA1c provides independent predictive information apart from baseline renal function, surgical complexity, and recognized clinical risk factors.

## 2. Methods

The study was approved by the institutional Ethics Committee (Protocol 370/23 May 2025).

This study included all patients who underwent cardiac and thoracic aorta surgeries at the cardiothoracic surgery clinic of “G. Papanikolaou” General Hospital in Thessaloniki from 1 November 2019 to 30 June 2021. Exclusion criteria were (i) age < 18 years, (ii) pregnancy, and (iii) no record of the patient’s HbA1c the last week before the surgery. Out of 805 patients who underwent elective cardiac and/or thoracic aorta surgeries during the specified period, 77 were excluded due to the absence of HbA1c measurement within the week prior to surgery.

Since there were no pregnant women or minors, the remaining 728 patients were included in the analysis.

Per institutional protocol, strict perioperative glucose management was applied, aiming to maintain blood glucose levels within a target range of 140–180 mg/dL.

The study’s variables were divided into three categories: (A) Baseline variables included patients’ age, body mass index (BMI), HbA1c, European System for Cardiac Operative Risk Evaluation II (EuroScore II) [13], ejection fraction (EF), creatinine and glomerular filtration rate (GFR) calculated with the modification of diet in renal disease (MDRD) equation [14], gender, smoking status [15], comorbidities (hypertension, diabetes mellitus, hyperlipidemia, peripheral vascular disease (PVD), chronic obstructive pulmonary disease (COPD), and atrial fibrillation (AF)), diabetes treatment, if any, previous cardiac surgery, and New York Heart Association (NYHA) class [16]. (B) Surgery-related variables included cardiopulmonary bypass (CPB) time, the number of transfused red blood cell (RBC) units, and the type of surgery, which included eight types of surgery: (i) coronary artery bypass graft surgery (CABG), (ii) CABG + valve surgery, (iii) CABG + thoracic aorta surgery, (iv) CABG + valve + thoracic aorta surgery, (v) valve surgery, (vi) valve + thoracic aorta surgery, (vii) thoracic aorta surgery, and (viii) other (atrial septal defect and ventricular septal defect closure surgery, heart tumor removal, septal myectomy in hypertrophic obstructive cardiomyopathy, surgical treatment for paravalvular leak, and left ventricle aneurysm repair). Furthermore, all CABG, valve, and thoracic aorta surgeries were grouped together in three separate groups. (C) Post-surgical variables included intra-aortic balloon pump (IABP) insertion, low cardiac output syndrome (LCOS), post-surgical AF, severe hemorrhage that led to re-surgery, transient ischemic attack (TIA) or stroke, sternal infection, pneumonia, septicemia, acute kidney injury (AKI) and its stage [17], need for continuous renal replacement therapy (CRRT), non-invasive ventilation (NIV) or re-intubation, duration of mechanical ventilation and ICU stay, and mortality.

Statistical analysis was performed using the SPSS (version 20 IBM-SPSS-statistical-software, Armonk, NY, USA). Continuous variables were presented as mean ± SD and categorical variables as number/total. *p* < 0.05 was accepted as statistically significant. Normality tests applying the Kolmogorov–Smirnov test were conducted to distinguish between parametric and non-parametric variables.

To examine the relationship between HbA1c and other categorical variables within the study, which have two possible outcomes, the independent samples *t*-test and the Mann–Whitney U-test were employed for parametric and non-parametric variables, respectively. For categorical variables with more than two potential outcomes, one-way ANOVA and the Kruskal–Wallis tests were utilized, with post hoc analyses being conducted using either the Bonferroni correction or the Mann–Whitney U-test for parametric and non-parametric variables, respectively.

In order to evaluate the relationship between HbA1c and the other continuous variables in the study, linear regression analysis was employed. Finally, to identify predictors of LCOS and mortality in relation to the other variables studied, univariate logistic regression analysis was initially performed. Subsequently, multivariate logistic regression analysis employing the backward likelihood ratio method was conducted between LCOS or mortality and the variables that demonstrated statistical significance in the univariate analysis.

## 3. Results

Table 1 shows all the study parameters and their relation to HbA1c. Among baseline parameters, BMI was significantly correlated with HbA1c (r = 0.08, *p* = 0.025), while in patients with NYHA class III, HbA1c was significantly higher compared to classes I and II (6.86 ± 1.60% vs. 6.13 ± 1.09% and 6.18 ± 1.18%, respectively, *p* = 0.001). There were no other baseline or surgical parameters that were related to HbA1c. As far as post-surgical parameters, HbA1c was significantly higher in patients who suffered from LCOS (7.05 ± 1.72% vs. 6.15 ± 1.13%, *p* = 0.013) and in those who did not survive (6.79 ± 1.50% vs. 6.15 ± 1.13%, *p* = 0.011).

Univariate logistic regression analysis showed that increasing HbA1c, EuroScore II, and NYHA class increased the risk for LCOS (OR (95% CI) = 1.53 (1.22–1.93), *p* < 0.001, OR (95% CI) = 1.10 (1.03–1.17), *p* = 0.004, and OR (95% CI) = 3.70 (2.04–6.70), *p* < 0.001, respectively), while increasing EF and GFR decreased the risk for LCOS (OR (95% CI) = 0.87 (0.83–0.90), *p* < 0.001 and OR (95% CI) = 0.98 (0.96–0.99), *p* = 0.003, respectively). Among surgical parameters, increasing CPB time and units of transfused RBCs increased the risk for LCOS (OR (95% CI) = 1.01 (1.00–1.02), *p* = 0.002 and OR (95% CI) = 1.34 (1.09–1.64), *p* = 0.006, respectively). Extensive surgeries that included simultaneous CABG, valve, and thoracic aorta operations increased the odds for LCOS (OR (95% CI) = 6.42 (1.32–31.32), *p* = 0.022), while all CABG surgeries grouped together also increased the odds for LCOS (OR (95% CI) = 11.79 (1.59–87.58), *p* = 0.016). As far as post-surgical parameters, AF also increased the risk for LCOS (OR (95% CI) = 3.84 (1.74–8.47), *p* = 0.001). Other factors, such as IABP insertion, pneumonia, AKI, CRRT, re-intubation, increasing hours of mechanical ventilation, and days in ICU, were also related significantly with LCOS; however, they were probably results and not causes of it (Table 2). Multivariate regression analysis showed that among all the parameters that were significantly related with LCOS in the univariate analysis, only increasing HbA1c and post-surgical AF increased the risk for LCOS (OR (95% CI) = 1.52 (1.09–2.12), *p* = 0.015 and OR (95% CI) = 4.42 (1.41–13.91), *p* = 0.011, respectively), while increasing baseline EF decreased the odds for LCOS (OR (95% CI) = 0.87 (0.83–0.92), *p* < 0.001). Post-surgical IABP insertion and AKI are also related to LCOS (OR (95% CI) = 8.75 (2.78–27.53), *p* < 0.001 and OR (95% CI) = 3.65 (1.08–12.31), *p* = 0.037) (Table 3).

The univariate analysis revealed a multitude of factors that were strongly connected to mortality. Higher values in BMI, HbA1c, EuroScore II, creatinine levels, and NYHA class, along with a history of prior cardiac surgery or baseline atrial fibrillation, were identified as significant factors increasing mortality odds. Conversely, raised ejection fraction and glomerular filtration rate were associated with reduced mortality odds.

Among surgical factors, extended CPB duration and higher volumes of transfused RBC units were also associated with an increased risk of mortality.

CABG was a predictor of favorable outcome; however, when CABG was combined with a valve or valve-plus-thoracic-aorta operation, the risk of mortality was significantly increased. Furthermore, when all valve or thoracic aorta procedures were combined, they also served as predictors of an adverse outcome.

Finally, among post-surgical factors, the emergence of LCOS, pneumonia, septicemia, AKI, increasing AKI stage or CRRT, IABP insertion, the need for NIV or re-intubation, and the increasing hours of mechanical ventilation or days in ICU were all predictors of significantly higher mortality (Table 4). However, in the multivariate analysis, among baseline characteristics, only increasing BMI, HbA1c, and EuroScore II were found to increase the risk of mortality (OR (95% CI) = 1.02 (1.00–1.03), *p* = 0.030, OR (95% CI) = 1.81 (1.19–2.74), *p* = 0.005, and OR (95% CI) = 1.22 (1.09–1.37), *p* = 0.001, respectively), while increasing GFR was found to decrease the risk for mortality (OR (95% CI) = 0.97 (0.94–1.00), *p* = 0.021). Increasing CPB time was the only significant predictor for unfavorable outcome among surgical factors (OR (95% CI) = 1.03 (1.02–1.04), *p* < 0.001), while the emergence of post-surgical septicemia or the need for CRRT or re-intubation were all strong predictors for mortality (OR (95% CI) = 71.11 (2.00–2527.41), *p* = 0.019, OR (95% CI) = 19.29 (2.18–170.54), *p* = 0.008, and OR (95% CI) = 36.70 (6.33–212.78), *p* < 0.001, respectively). Finally, increasing hours of mechanical ventilation were also predicting significantly higher odds for mortality (OR (95% CI) = 1.06 (1.03–1.09), *p* < 0.001); however, increasing days of stay in the ICU predicted significantly lower odds for mortality (OR (95% CI) = 0.71 (0.58–0.88), *p* = 0.002) (Table 5).

## 4. Discussion

In this single-center retrospective cohort study of 728 patients who underwent elective cardiac and/or thoracic aortic surgery, higher preoperative HbA1c levels were found to be independently associated with an increased risk of postoperative low cardiac output syndrome (LCOS) and in-hospital mortality.

The associations remained significant even after accounting for established risk factors, including EuroScore II, renal function, surgical complexity, and baseline left ventricular ejection fraction (EF). Our results indicate a potential additional prognostic role of HbA1c beyond traditional cardiac surgical risk stratification tools, warranting further evaluation in prospective interventional studies.

The relationship between chronic dysglycemia and negative postoperative outcomes has garnered increasing attention. Elevated HbA1c levels signal prolonged hyperglycemia, which may lead to microvascular dysfunction, impaired myocardial energetics, oxidative stress, and endothelial injury [18,19,20,21].

When these mechanisms are combined, they may heighten the risk of postoperative myocardial dysfunction and ischemia–reperfusion injury [22].

Our observation that patients who developed LCOS exhibited markedly elevated HbA1c levels substantiates this pathophysiological rationale.

In cardiac surgery, multiple studies have established an association between preoperative HbA1c levels and postoperative outcomes. Halkos et al. identified a correlation between HbA1c levels above 8% and increased morbidity and mortality rates in patients who underwent coronary artery bypass grafting (CABG). The study indicated that an A1c level exceeding 8.6% correlated with a fourfold increase in mortality [23].

Wang et al. published a meta-analysis demonstrating that elevated preoperative HbA1c levels are associated with heightened risks of surgical site infection, renal failure, and myocardial infarction, along with prolonged hospital stays in diabetic patients undergoing coronary artery disease surgery. Additionally, higher mortality and renal failure rates have been observed in nondiabetic patients [24].

Significant variability in the definition of elevated HbA1c was observed, highlighting the necessity for large, high-quality randomized controlled trials. A meta-analysis conducted by Jia Zheng et al. identified a potential link between elevated HbA1c levels and increased risks of all-cause mortality, myocardial infarction, and stroke in diabetic patients undergoing coronary artery bypass grafting (CABG).

Nevertheless, further clinical research with larger sample sizes and extended follow-up periods is essential to support these findings [25]. Additionally, Finger et al. demonstrated that elevated HbA1c levels serve as predictors of postoperative renal impairment and prolonged hospitalization after cardiac surgery [26]. Higher HbA1c levels in nondiabetic patients were associated with increased adverse outcomes following CABG, as reported by Liu et al. [27]. Elevated preoperative HbA1c levels also had a negative impact on late mortality after cardiac surgery, according to a meta-analysis by Corazzari et al. [7].

Some studies have challenged the independent prognostic significance of HbA1c, indicating that diabetes status and intraoperative glycemic management may have a stronger correlation with clinical outcomes [28,29].

The observed heterogeneity across studies may arise from variations in patient demographics, differing definitions of hyperglycemia, the methods used to adjust for confounding variables, and differences in surgical techniques.

Our research adds to the ongoing debate by demonstrating that HbA1c serves as a valuable independent predictor of low cardiac output syndrome (LCOS) and mortality among diverse populations undergoing cardiac and thoracic aortic surgery. This conclusion remains valid even after accounting for EuroSCORE II, a widely accepted metric for evaluating operative risk.

The inclusion of valve and aortic procedures in our cohort, unlike previous studies that mainly concentrated on patients undergoing coronary artery bypass grafting, enhances the generalizability of our findings.

Moreover, LCOS has been inadequately studied in previous research, despite its significant impact on perioperative morbidity and mortality.

The observed association between HbA1c and LCOS in the multivariable analysis indicates that inadequate preoperative glycemic control might increase the risk of postoperative hemodynamic instability. This effect may be influenced by factors such as impaired myocardial reserve, autonomic dysfunction, or a reduced ability to tolerate the systemic inflammatory response that results from cardiopulmonary bypass.

The significant correlations found between mortality and postoperative complications—such as septicemia, reintubation, prolonged mechanical ventilation, and the need for renal replacement therapy—are consistent with established predictors of adverse outcomes in cardiac surgery [30,31].

HbA1c’s continuing value as an independent predictor, even after accounting for these features, emphasizes its potential as a simple, easily accessible biomarker for improved preoperative risk assessment.

The apparent link between a longer ICU stay and lower mortality may be attributed to survivorship bias. Specifically, patients who survive often require extended supportive care, whereas those who do not survive frequently pass away during the initial hospitalization.

These study results have important clinical implications. HbA1c is an inexpensive, widely available, and noninvasive marker that may aid preoperative risk stratification in elective cardiac surgery. Moreover, as an adjustable variable, HbA1c offers a potential target for preoperative glycemic management; however, this strategy necessitates validation via prospective interventional trials. Patients with elevated HbA1c levels may need more intensive perioperative monitoring and more effective preventative strategies to address potential complications, such as acute renal damage and infection.

Several limitations must be recognized. The retrospective design restricts the ability to establish causal relationships, and there is also a risk of residual confounding.

The analysis did not include postoperative glycemic values, intraoperative glucose management, or the application of insulin protocols; incorporating these data could have facilitated a more thorough assessment of the relationship between chronic and acute glycemic control.

The single-center design may further limit the generalizability of the findings to institutions with differing patient populations or perioperative management strategies. Finally, certain postoperative variables associated with mortality, such as septicemia or the need for continuous renal replacement therapy, may function as mediators rather than independent predictors.

Our findings, despite certain limitations, add to the growing body of evidence that chronic glycemic status, as indicated by HbA1c levels, has a significant impact on postoperative outcomes in cardiac surgery.

Research data suggests that HbA1c should be incorporated into risk stratification models and may serve as a target for optimizing preoperative care in elective procedures.

## 5. Conclusions

In this retrospective cohort of patients undergoing elective cardiac and thoracic aortic surgery, elevated preoperative HbA1c levels were independently associated with an increased risk of postoperative low cardiac output syndrome and in-hospital mortality. After adjustment for established clinical risk scores, renal function, and surgical complexity, HbA1c remained independently associated with outcomes, indicating a potential incremental prognostic value beyond conventional risk assessment tools. The clinical utility of incorporating HbA1c into preoperative evaluation and risk optimization strategies requires further investigation in prospective interventional studies.

## Figures and Tables

**Table 1:** Study parameters and their relation to HbA1c.

Parameter (Mean ± s.d.)		B (95% C.I.)	R	*p* (Value)
Baseline				
Age (66.28 ± 9.65 years)		−0.01 (−0.02–0.00)	−0.05	0.17
BMI (29.58 ± 16.15 Kg/m^2^)		0.01 (0.00–0.01)	0.08	**0.025**
HbA1c (6.18 ± 1.16%)		n/a	n/a	n/a
EuroScore II (2.44 ± 3.17)		−0.00 (−0.20–0.20)	0.00	0.99
EF (54.48 ± 10.69%)		−0.59 (−1.26–0.08)	−0.06	0.09
Creatinine (1.09 ± 0.93 mg/dL)		−0.03 (−0.09–0.02)	−0.04	0.25
GFR (79.85 ± 24.38 mL/min/1.73 m^2^)		0.88 (−0.65–2.41)	0.04	0.26
Surgery				
CPB time (108.27 ± 47.37 min)		0.31 (−2.66–3.28)	0.01	0.84
RBC transfusion (1.53 ± 1.44 Units)		−0.07 (−0.16–0.02)	−0.06	0.13
Post-surgery				
Hours of mechanical ventilation (13.69 ± 17.55)		0.72 (−0.38–1.82)	0.05	0.20
Days in ICU (2.33 ± 2.89)		−0.03 (−0.21–0.15)	−0.01	0.75
**Parameter (N/T, %)**		**Mean ± s.d.**		***p* (Value)**
Baseline				
Gender	Male (572/728, 78.6%)	6.19 ± 1.16		0.92
	Female (156/728, 21.4%)	6.17 ± 1.18		
Ever smoker	No (431/728, 59.2%)	6.20 ± 1.17		0.55
	Yes (297/728, 40.8%)	6.15 ± 1.45		
Current smoker	No (532/728, 73.1%)	6.21 ± 1.21		0.39
	Yes (196/728, 26.9%)	6.12 ± 1.02		
Hypertension	No (223/728, 30.6%)	6.18 ± 1.13		0.99
	Yes (505/728, 69.4%)	6.18 ± 1.18		
Diabetes mellitus	No (482/728, 66.2%)	6.16 ± 1.15		0.54
	Yes (246/728, 33.8%)	6.22 ± 1.18		
Diabetes treatment	Diet (1/246, 0.4%)	5.90 ± n/a		0.49
	Tablets (169/246, 68.7%)	6.16 ± 1.12		
	Insulin (76/246, 30.9%)	6.35 ± 1.31		
Hyperlipidemia	No (302/728, 41.5%)	6.09 ± 1.06		0.06
	Yes (426/728, 58.5%)	6.25 ± 1.23		
PVD	No (616/728, 84.6%)	6.20 ± 1.19		0.33
	Yes (112/728, 15.4%)	6.08 ± 1.01		
COPD	No (679/728, 93.3%)	6.18 ± 1.16		0.87
	Yes (49/728, 6.7%)	6.21 ± 1.24		
Previous cardiac surgery	No (704/728, 96.7%)	6.19 ± 1.17		0.60
	Yes (24/728, 3.3%)	6.06 ± 1.07		
NYHA class	I (416/728, 57.1%)	6.13 ± 1.09		**0.001**
	II (276/728, 37.9%)	6.18 ± 1.18		
	III (36/728, 4.9%)	6.86 ± 1.60		
	IV (0/728, 0.0%)	n/a		
AF	No (628/728, 86.3%)	6.20 ± 1.17		0.36
	Yes (100/728, 13.7%)	6.08 ± 1.14		
Surgery				
CABG	No (333/728, 45.7%)	6.13 ± 1.12		0.28
	Yes (395/728, 54.3%)	6.23 ± 1.20		
CABG + Valve surgery	No (646/728, 88.7%)	6.20 ± 1.17		0.30
	Yes (82/728, 11.3%)	6.06 ± 1.12		
CABG + Thoracic Aorta surgery	No (714/728, 98.1%)	6.18 ± 1.17		0.88
	Yes (14/728, 1.9%)	6.14 ± 0.89		
CABG + Valve surgery + Thoracic Aorta surgery	No (717/728, 98.5%)	6.18 ± 1.17		0.98
	Yes (11/728, 1.5%)	6.17 ± 0.90		
Valve surgery	No (591/728, 81.2%)	6.21 ± 1.19		0.15
	Yes (137/728, 18.8%)	6.05 ± 1.04		
Valve surgery + Thoracic Aorta surgery	No (710/728, 97.5%)	6.17 ± 1.15		0.14
	Yes (18/728, 2.5%)	6.74 ± 1.57		
Thoracic Aorta surgery	No (676/728, 92.9%)	6.18 ± 1.17		0.91
	Yes (52/728, 7.1%)	6.17 ± 1.10		
Other	No (709/728, 97.4%)	6.18 ± 1.15		0.58
	Yes (19/728, 2.6%)	6.34 ± 1.47		
All CABG	No (226/728, 31.0%)	6.16 ± 1.15		0.69
	Yes (502/728, 69.0%)	6.19 ± 1.17		
All Valve surgeries	No (480/728, 65.9%)	6.22 ± 1.19		0.22
	Yes (248/728, 34.1%)	6.11 ± 1.11		
All Thoracic Aorta surgeries	No (633/728, 87.0%)	6.17 ± 1.16		0.42
	Yes (95/728, 13.0%)	6.27 ± 1.16		
Post-surgery				
IABP insertion	No (678/728, 93.1%)	6.17 ± 1.14		0.23
	Yes (50/728, 6.9%)	6.37 ± 1.40		
Hemorrhage	No (717/728, 98.5%)	6.19 ± 1.17		0.51
	Yes (11/728, 1.5%)	5.96 ± 0.73		
LCOS	No (702/728, 96.4%)	6.15 ± 1.13		**0.013**
	Yes (26/728, 3.6%)	7.05 ± 1.72		
AF	No (570/728, 78.3%)	6.15 ± 1.13		0.13
	Yes (158/728, 21.7%)	6.31 ± 1.26		
TIA or stroke	No (721/728, 99.0%)	6.19 ± 1.17		0.58
	Yes (7/728, 1.0%)	5.94 ± 0.57		
Sternal infection	No (725/728, 99.6%)	6.18 ± 1.16		0.57
	Yes (3/728, 0.4%)	5.80 ± 0.87		
Pneumonia	No (719/728, 98.8%)	6.18 ± 1.17		0.96
	Yes (9/728, 1.2%)	6.20 ± 0.70		
Septicemia	No (720/728, 98.9%)	6.18 ± 1.17		0.96
	Yes (8/728, 1.1%)	6.16 ± 0.59		
AKI	No (604/728, 83.0%)	6.16 ± 1.12		0.28
	Yes (124/728, 17.0%)	6.30 ± 1.36		
AKI stage	I (63/124, 50.8%)	6.21 ± 1.33		0.35
	II (30/124, 24.2%)	6.17 ± 1.39		
	III (31/124, 25.0%)	6.61 ± 1.40		
CRRT	No (704/728, 96.7%)	6.17 ± 1.15		0.09
	Yes (24/728, 3.3%)	6.58 ± 1.44		
NIV	No (691/728, 94.9%)	6.18 ± 1.16		0.91
	Yes (37/728, 5.1%)	6.16 ± 1.17		
Re-intubation	No (709/728, 97.4%)	6.18 ± 1.17		0.66
	Yes (19/728, 2.6%)	6.30 ± 0.85		
Survival	Yes (689/728, 94.6%)	6.15 ± 1.13		**0.011**
	No (39/728, 5.4%)	6.79 ± 1.50		

*p* values shown with Bold indicate statistical significance. HbA1c = Hemoglobin A1c, s.d. = standard deviation, C.I. = confidence interval, BMI = body mass index, Kg = kilograms, m = meters, n/a = not applicable, EuroScore II = European System for Cardiac Operative Risk Evaluation II, EF = ejection fraction, mg = milligrams, dL = deciliter, GFR = glomerular filtration rate, mL = milliliters, min = minute, CPB = cardiopulmonary bypass, RBCs = red blood cells, ICU = intensive care unit, N = number, T = total, PVD = peripheral vascular disease, COPD = chronic obstructive pulmonary disease, NYHA = New York Heart Association, AF = atrial fibrillation, CABG = coronary artery bypass graft surgery, IABP = intra-aortic balloon pump, LCOS = low cardiac output syndrome, TIA = transient ischemic attack, AKI = acute kidney injury, CRRT = continuous renal replacement therapy, NIV = non-invasive ventilation.

**Table 2:** Univariate logistic regression analysis between LCOS and the other study parameters.

Parameter	OR (95% C.I.)	*p* (Value)
Baseline		
Age (years)	1.02 (0.98–1.07)	0.32
BMI (Kg/m^2^)	0.97 (0.90–1.06)	0.53
HbA1c (%)	1.53 (1.22–1.93)	**<0.001**
EuroScore II	1.10 (1.03–1.17)	**0.004**
EF (%)	0.87 (0.83–0.90)	**<0.001**
Creatinine (mg/dL)	1.13 (0.87–1.47)	0.36
GFR (mL/min/1.73 m^2^)	0.98 (0.96–0.99)	**0.003**
Gender (Female)	0.14 (0.02–1.05)	0.06
Ever smoker (Yes)	1.25 (0.57–2.75)	0.57
Current smoker (Yes)	1.22 (0.52–2.84)	0.65
Hypertension (Yes)	0.70 (0.31–1.56)	0.38
Diabetes mellitus (Yes)	1.71 (0.78–3.77)	0.18
Diabetes treatment (Insulin)	1.63 (0.50–5.31)	0.42
Hyperlipidemia (Yes)	0.70 (0.32–1.53)	0.37
PVD (Yes)	1.69 (0.66–4.30)	0.27
COPD (Yes)	1.86 (0.54–6.43)	0.33
Previous cardiac surgery (Yes)	0.00 (n/a)	1.00
NYHA class	3.70 (2.04–6.70)	**<0.001**
AF (Yes)	1.52 (0.56–4.13)	0.41
Surgery		
CPB time (minutes)	1.01 (1.00–1.02)	**0.002**
RBC transfusion (Units)	1.34 (1.09–1.64)	**0.006**
CABG (Yes)	1.62 (0.71–3.68)	0.25
CABG + Valve surgery (Yes)	2.47 (0.96–6.34)	0.06
CABG + Thoracic Aorta surgery (Yes)	0.00 (n/a)	1.00
CABG + Valve surgery + Thoracic Aorta surgery (Yes)	6.42 (1.32–31.32)	**0.022**
Valve surgery (Yes)	0.17 (0.02–1.24)	0.08
Valve surgery + Thoracic Aorta surgery (Yes)	0.00 (n/a)	1.00
Thoracic Aorta surgery (Yes)	0.00 (n/a)	1.00
Other (Yes)	0.00 (n/a)	1.00
All CABG (Yes)	11.79 (1.59–87.58)	**0.016**
All Valve surgeries (Yes)	1.03 (0.45–2.34)	0.95
All Thoracic Aorta surgeries (Yes)	0.42 (0.13–2.35)	0.42
Post-surgery		
IABP insertion (Yes)	38.29 (15.88–92.35)	**<0.001**
Hemorrhage (Yes)	0.00 (n/a)	1.00
AF (Yes)	3.84 (1.74–8.47)	**0.001**
TIA or stroke (Yes)	0.00 (n/a)	1.00
Sternal infection (Yes)	0.00 (n/a)	1.00
Pneumonia (Yes)	8.27 (1.63–41.95)	**0.011**
Septicemia (Yes)	3.97 (0.47–33.52)	0.21
AKI (Yes)	10.50 (4.56–24.18)	**<0.001**
AKI stage	1.38 (0.76–2.51)	0.29
CRRT (Yes)	8.56 (2.92–25.12)	**<0.001**
NIV (Yes)	1.59 (0.36–6.99)	0.54
Re-intubation (Yes)	5.59 (1.52–20.55)	**0.010**
Hours of mechanical ventilation	1.03 (1.02–1.04)	**<0.001**
Days in ICU	1.14 (1.08–1.22)	**<0.001**

*p* values shown with Bold indicate statistical significance. LCOS = low cardiac output syndrome, OR = odds ratio, C.I. = confidence interval, BMI = body mass index, Kg = kilograms, m = meters, HbA1c = Hemoglobin A1c, EuroScore II = European System for Cardiac Operative Risk Evaluation II, EF = ejection fraction, mg = milligrams, dL = deciliter, GFR = glomerular filtration rate, mL = milliliters, min = minute, PVD = peripheral vascular disease, COPD = chronic obstructive pulmonary disease, n/a = not applicable, NYHA = New York Heart Association, AF = atrial fibrillation, CPB = cardiopulmonary bypass, RBCs = red blood cells, CABG = coronary artery bypass graft surgery, IABP = intra-aortic balloon pump, TIA = transient ischemic attack, AKI = acute kidney injury, CRRT = continuous renal replacement therapy, NIV = non-invasive ventilation, ICU = intensive care unit.

**Table 3:** Multivariate logistic regression analysis between LCOS and the significant predictors of the univariate analysis.

Predictor	OR (95% C.I.)	*p* (Value)
Baseline		
HbA1c (%)	1.52 (1.09–2.12)	**0.015**
EuroScore II	1.06 (0.87–1.30)	0.55
EF (%)	0.87 (0.83–0.92)	**<0.001**
GFR (mL/min/1.73 m^2^)	1.00 (0.97–1.02)	0.82
NYHA class	1.09 (0.39–3.06)	0.87
Surgery		
CPB time (minutes)	1.00 (0.99–1.02)	0.51
RBC transfusion (Units)	1.14 (0.85–1.53)	0.40
CABG + Valve surgery + Thoracic Aorta surgery (Yes)	0.33 (0.02–4.93)	0.42
All CABG (Yes)	3.68 (0.39–34.44)	0.25
Post-surgery		
IABP insertion (Yes)	8.75 (2.78–27.53)	**<0.001**
AF (Yes)	4.42 (1.41–13.91)	**0.011**
Pneumonia (Yes)	0.79 (0.05–12.46)	0.87
AKI (Yes)	3.65 (1.08–12.31)	**0.037**
CRRT (Yes)	5.46 (0.98–30.38)	0.05
Re-intubation (Yes)	0.76 (0.12–5.09)	0.78
Hours of mechanical ventilation	0.99 (0.96–1.01)	0.33
Days in ICU	1.08 (0.97–1.21)	0.15

*p* values shown with Bold indicate statistical significance. LCOS = low cardiac output syndrome, OR = odds ratio, C.I. = confidence interval, HbA1c = Hemoglobin A1c, EuroScore II = European System for Cardiac Operative Risk Evaluation II, EF = ejection fraction, GFR = glomerular filtration rate, mL = milliliters, min = minute, m = meters, NYHA = New York Heart Association, CPB = cardiopulmonary bypass, RBCs = red blood cells, CABG = coronary artery bypass graft surgery, IABP = intra-aortic balloon pump, AF = atrial fibrillation, AKI = acute kidney injury, CRRT = continuous renal replacement therapy, ICU = intensive care unit.

**Table 4:** Univariate logistic regression analysis between mortality and the other study parameters.

Parameter	OR (95% C.I.)	*p* (Value)
Baseline		
Age (years)	1.00 (0.96–1.03)	0.79
BMI (Kg/m^2^)	1.01 (1.00–1.02)	**0.046**
HbA1c (%)	1.41 (1.14–1.73)	**0.001**
EuroScore II	1.25 (1.16–1.34)	**<0.001**
EF (%)	0.96 (0.94–0.99)	**0.007**
Creatinine (mg/dL)	1.24 (1.03–1.49)	**0.026**
GFR (mL/min/1.73 m^2^)	0.97 (0.96–0.98)	**<0.001**
Gender (Female)	1.68 (0.83–3.40)	0.15
Ever smoker (Yes)	0.71 (0.36–1.41)	0.33
Current smoker (Yes)	1.07 (0.52–2.19)	0.85
Hypertension (Yes)	1.76 (0.80–3.89)	0.16
Diabetes mellitus (Yes)	0.86 (0.43–1.74)	0.68
Diabetes treatment (Insulin)	2.33 (0.73–7.47)	0.16
Hyperlipidemia (Yes)	0.91 (0.48–1.75)	0.78
PVD (Yes)	1.22 (0.52–2.83)	0.65
COPD (Yes)	2.16 (0.80–5.79)	0.13
Previous cardiac surgery (Yes)	3.82 (1.24–11.79)	**0.020**
NYHA class	4.74 (2.83–7.93)	**<0.001**
AF (Yes)	2.30 (1.08–4.87)	**0.030**
Surgery		
CPB time (minutes)	1.03 (1.02–1.03)	**<0.001**
RBC transfusion (Units)	1.33 (1.11–1.59)	**0.002**
CABG (Yes)	0.27 (0.13–0.57)	**0.001**
CABG + Valve surgery (Yes)	2.53 (1.16–5.54)	**0.020**
CABG + Thoracic Aorta surgery (Yes)	1.37 (0.17–10.74)	0.77
CABG + Valve surgery + Thoracic Aorta surgery (Yes)	16.74 (4.87–57.60)	**<0.001**
Valve surgery (Yes)	1.12 (0.50–2.49)	0.78
Valve surgery + Thoracic Aorta surgery (Yes)	0.00 (n/a)	1.00
Thoracic Aorta surgery (Yes)	1.53 (0.52–4.47)	0.44
Other (Yes)	2.14 (0.48–9.60)	0.32
All CABG (Yes)	0.79 (0.41–1.56)	0.50
All Valve surgeries (Yes)	2.65 (1.38–5.09)	**0.003**
All Thoracic Aorta surgeries (Yes)	2.45 (1.15–5.21)	**0.020**
Post-surgery		
LCOS (Yes)	6.08 (2.29–16.16)	**<0.001**
IABP insertion (Yes)	5.60 (2.55–12.28)	**<0.001**
Hemorrhage (Yes)	4.08 (0.85–19.58)	0.08
AF (Yes)	1.45 (0.70–2.98)	0.31
TIA or stroke (Yes)	3.00 (0.35–25.51)	0.32
Sternal infection (Yes)	0.00 (n/a)	1.00
Pneumonia (Yes)	5.27 (1.06–26.24)	**0.043**
Septicemia (Yes)	33.63 (7.72–146.58)	**<0.001**
AKI (Yes)	15.72 (7.58–32.63)	**<0.001**
AKI stage	8.58 (3.95–18.63)	**<0.001**
CRRT (Yes)	97.57 (35.15–270.82)	**<0.001**
NIV (Yes)	3.02 (1.11–8.24)	**0.031**
Re-intubation (Yes)	33.44 (12.48–89.64)	**<0.001**
Hours of mechanical ventilation	1.05 (1.04–1.06)	**<0.001**
Days in ICU	1.14 (1.07–1.21)	**<0.001**

*p* values shown with Bold indicate statistical significance. OR = odds ratio, C.I. = confidence interval, BMI = body mass index, Kg = kilograms, m = meters, HbA1c = Hemoglobin A1c, EuroScore II = European System for Cardiac Operative Risk Evaluation II, EF = ejection fraction, mg = milligrams, dL = deciliter, GFR = glomerular filtration rate, mL = milliliters, min = minute, PVD = peripheral vascular disease, COPD = chronic obstructive pulmonary disease, NYHA = New York Heart Association, AF = atrial fibrillation, CPB = cardiopulmonary bypass, RBCs = red blood cells, CABG = coronary artery bypass graft surgery, n/a = not applicable, LCOS = low cardiac output syndrome, IABP = intra-aortic balloon pump, TIA = transient ischemic attack, AKI = acute kidney injury, CRRT = continuous renal replacement therapy, NIV = non-invasive ventilation, ICU = intensive care unit.

**Table 5:** Multivariate logistic regression analysis between mortality and the significant predictors of the univariate analysis.

Predictor	OR (95% C.I.)	*p* (Value)
Baseline		
BMI (Kg/m^2^)	1.02 (1.00–1.03)	**0.030**
HbA1c (%)	1.81 (1.19–2.74)	**0.005**
EuroScore II	1.22 (1.09–1.37)	**0.001**
EF (%)	1.00 (0.93–1.07)	0.96
Creatinine (mg/dL)	1.24 (0.64–2.39)	0.52
GFR (mL/min/1.73 m^2^)	0.97 (0.94–1.00)	**0.021**
Previous cardiac surgery (Yes)	0.08 (0.00–2.64)	0.16
NYHA class	1.47 (0.44–4.95)	0.53
AF (Yes)	1.61 (0.35–7.47)	0.54
Surgery		
CPB time (minutes)	1.03 (1.02–1.04)	**<0.001**
RBC transfusion (Units)	0.63 (0.40–1.01)	0.06
CABG (Yes)	1.41 (0.24–8.14)	0.70
CABG + Valve surgery (Yes)	0.32 (0.05–2.03)	0.23
CABG + Valve surgery + Thoracic Aorta surgery (Yes)	0.21 (0.00–1254.33)	0.73
All Valve surgeries (Yes)	5.76 (0.14–241.46)	0.36
All Thoracic Aorta surgeries (Yes)	0.10 (0.01–1.09)	0.06
Post-surgery		
LCOS (Yes)	0.95 (0.05–18.28)	0.97
IABP insertion (Yes)	1.42 (0.21–9.43)	0.72
Pneumonia (Yes)	0.00 (n/a)	1.00
Septicemia (Yes)	71.11 (2.00–2527.41)	**0.019**
AKI (Yes)	0.68 (0.12–3.94)	0.68
CRRT (Yes)	19.29 (2.18–170.54)	**0.008**
NIV (Yes)	0.12 (0.01–1.92)	0.13
Re-intubation (Yes)	36.70 (6.33–212.78)	**<0.001**
Hours of mechanical ventilation	1.06 (1.03–1.09)	**<0.001**
Days in ICU	0.71 (0.58–0.88)	**0.002**

*p* values shown with Bold indicate statistical significance. OR = odds ratio, C.I. = confidence interval, BMI = body mass index, Kg = kilograms, m = meters, HbA1c = Hemoglobin A1c, EuroScore II = European System for Cardiac Operative Risk Evaluation II, EF = ejection fraction, mg = milligrams, dL = deciliter, GFR = glomerular filtration rate, mL = milliliters, min = minute, NYHA = New York Heart Association, AF = atrial fibrillation, CPB = cardiopulmonary bypass, RBCs = red blood cells, CABG = coronary artery bypass graft surgery, LCOS = low cardiac output syndrome, IABP = intra-aortic balloon pump, n/a = not applicable, AKI = acute kidney injury, CRRT = continuous renal replacement therapy, NIV = non-invasive ventilation, ICU = intensive care unit.

## Data Availability

The data presented in this study are available from the corresponding author upon reasonable request.
